# Sociodemographic variations in healthcare service use among people with HIV in the UK

**DOI:** 10.1371/journal.pone.0354425

**Published:** 2026-09-15

**Authors:** Fumiyo Nakagawa, Valerie Delpech, Colette J. Smith, Annegret Pelchen-Matthews, Adamma Aghaizu, Clare Humphreys, Victoria Tittle, Lisa Southon, Alex Sparrowhawk, Harun Tulunay, Veronique Martin, Hannah Kitt, Carole Kelly, Janey Sewell, Alison Rodger, Fiona C. Lampe, for the Positive Voices 2022 Study Team

**Affiliations:** 1 Institute for Global Health, University College London, London, United Kingdom; 2 Mid-North Coast NSW Health, Coffs Harbour, New South Wales, Australia; 3 UK Health Security Agency, London, United Kingdom; 4 Chelsea and Westminster Hospital NHS Foundation Trust, London, United Kingdom; 5 Manchester University NHS Trust, Manchester, United Kingdom; 6 George House Trust, Manchester, United Kingdom; 7 Positively UK, London, United Kingdom; 8 Royal Free London NHS Foundation Trust, London, United Kingdom; University of Oxford, UNITED KINGDOM OF GREAT BRITAIN AND NORTHERN IRELAND

## Abstract

**Objectives:**

People with HIV on effective treatment can expect a normal life expectancy, though they may experience increased prevalence of other chronic health conditions. We assessed among people with HIV, non-HIV health care usage and variation by sociodemographic and lifestyle factors.

**Methods:**

Positive Voices 2022 is a national UK questionnaire study of people with HIV (April 2022-March 2023). Participants self-reported on use of a range of health services. Associations of sociodemographic and lifestyle factors with five specific services were assessed using modified Poisson regression. Prevalence ratios(aPRs) adjusted for age, demographic group and year of HIV diagnosis and 95% confidence intervals [95%CIs] are given.

**Results:**

Among 4451 people with healthcare use data, 79.0% attended their GP service in the past year, and 54.9%, 46.2%, 21.0%, and 10.0% attended dental, sexual health, accident and emergency (A&E) and mental health services, respectively. Use varied across sociodemographic groups. Older people were less likely to use mental health and sexual health services, but more likely to use GP and dentists. Black African men, other heterosexual men and Black African women were significantly less likely to use mental health services: aPR 0.19 95%CI[0.09–0.43], aPR 0.49 95%CI[0.31–0.78] and aPR 0.48 95%CI[0.34–0.68] respectively, compared to gay/bisexual/other men-who-have-sex-with-men. Participants without money for basic needs were much more likely to use mental health services (aPR 3.41, 95%CI[2.54–4.59] versus enough money) and A&E (aPR 2.00, 95%CI[1.65–2.45]), but less likely to use dentists (aPR 0.84 95%CI[0.73–0.96]). Obesity, smoking and comorbidities were also associated with higher use of A&E and mental health services.

**Conclusion:**

Healthcare service use among people with HIV varies across sociodemographic groups. Results suggest the need for interventions and policies to improve physical and mental health and access to timely care among people with socioeconomic disadvantage and to enable greater access to mental health services for Black African people.

## Introduction

Improvements in HIV care mean that people with HIV can now expect to have a normal life expectancy when diagnosed promptly and treated successfully [1]. In England, 98% of people diagnosed with HIV are receiving care and are on treatment, and 98% of people on treatment are virally suppressed [2,3]. National guidelines now recommend that for most virologically suppressed people on antiretroviral therapy (ART), attendance at specialist HIV clinics is required annually and HIV viral load checks carried out every six to 12 months [4]. However, over time the average age of the population of people with HIV has risen: in 2023 more than half of those with diagnosed HIV in the UK, 51%, were aged over 50 years [3] compared to 27% in 2013. This has resulted in an increase in comorbidities and other long-term conditions associated with ageing, which impacts on use of health services outside of HIV. Furthermore, evidence suggests that people with HIV have an increased prevalence of comorbidities when compared to age-matched HIV-negative populations, further impacting on need for health services [5].

In addition to age and comorbidities, socioeconomic factors influence use of healthcare. Healthcare in the UK, including care for HIV and access to ART is universal and free at the point of use through the National Health Service (NHS). A large body of research undertaken in high-income settings, including those with universal healthcare has demonstrated that socioeconomic factors are key drivers of both healthcare utilisation and health outcomes in the general population [6–10], with lower socioeconomic status being a strong predictor of the occurrence of physical and mental health disorders [11]. To our knowledge, few studies have assessed the effect of sociodemographic factors on differing use of healthcare services among people with HIV. Compared to the general population, people with HIV may be disproportionately affected by adverse socioeconomic factors such as financial hardship and lack of social support [12]. Furthermore, socioeconomic disadvantage has been shown to be associated with poorer ART adherence and virological outcomes among people with HIV [13]. Therefore, socioeconomic factors are likely to be important determinants of healthcare usage among people with HIV. Adverse lifestyle factors such as smoking, high alcohol intake, overweight and obesity are also strongly associated with increased morbidity, mortality and use of health services in the general population [14–17] and may also be important factors influencing wider health service usage among people with HIV.

Knowledge of levels of use of health services outside of HIV care and understanding of how this varies according to sociodemographic and lifestyle factors could provide insight into the wider healthcare needs of people with HIV. This paper describes self-reported use of healthcare services outside of standard HIV care among people with HIV in the UK, and assesses variation by sociodemographic factors (demographic group, age, year of HIV diagnosis, socioeconomic status), comorbidity and lifestyle factors (body mass index, smoking and alcohol intake) using data from a large cross-sectional survey of people with HIV in the UK.

## Methods

### Study design

We used data from Positive Voices 2022 (PV2022), a national cross-sectional survey conducted between 11^th^ April 2022 and 31^st^ March 2023 in 101 HIV clinics in England, Scotland and Wales. Participants were initially selected from participating clinics using random sampling to provide a representative sample of people with HIV accessing care in the UK. HARS, the national surveillance databased consisting of pseudonymized data on demographic and clinical characteristics of people with HIV held at the UK Health Security Agency, was used as a sampling frame which meant that the questionnaire data were linked to clinical data on ART, HIV viral load, and CD4 count. During the study recruitment period, the monthly study logs suggested that several sites were under-recruiting, largely resulting from fewer face-to-face clinic appointments due to the Covid-19 pandemic restrictions as well as the 2022 mpox outbreak at the time. Therefore, sequential recruitment was used from December 2022 in 14 London clinics to increase participant numbers. This entailed a recruitment strategy whereby a site could approach any eligible individual attending the clinic to invite them to participate in the study, instead of contacting participants from their pre-selected sample list. Participants were provided with an information sheet about the study and consent was implied by questionnaire completion. Further details on survey methodology have been described previously [18].

Participants completed a confidential self-administered questionnaire including demographic and socioeconomic information, HIV-related factors, health and wellbeing and lifestyle factors. Details of variables used in this analysis are given below.

### NHS and support service use

Participants were asked questions about their use of NHS, social care and support services (including other charity or voluntary organisations) in the previous year. These included questions related to primary care use: “Are you registered with a general practice doctor (GP)?” and “Does your GP know your HIV status?”. In addition, they were asked “whether you have had any contact with the services listed below in the last year and, if so, the number of times”; this included online health services and consultations. Responses required for each healthcare service were either “Yes” or “No” and “If yes, number of contacts”. Participants were asked to give an estimate of the number of contacts if they were unsure. Listed health services included GP (doctor or practice nurse), dentist, use of the NHS non-urgent telephone helpline (NHS direct/111), ambulance/paramedic, sexual health (also known as genito-urinary medicine, GUM) clinic, accident & emergency (A&E, including minor injuries department, NHS walk-in centre and urgent care), overnight stay on a hospital ward, physiotherapy, mental health services, HIV support services (charity or voluntary organisation), occupational therapy, antenatal clinic, substance misuse services and social services. Participants were able to list up to three additional specialist or hospital outpatient services.

### Sociodemographic and lifestyle factors

Age was grouped into five categories: 18–34, 35–44, 45–54, 55–64, 65 + years. Participants were classified into one of seven demographic groups as defined by their gender, ethnicity, and sexual orientation: GBMSM (gay, bisexual and other men-who-have-sex-with-men), Black African heterosexual men, other heterosexual men, Black African women, other women, non-binary or other gender, or undisclosed gender/sexuality. Year of HIV diagnosis was grouped into 1995 or earlier, 1996–2001, 2002–2007, 2008–2013, 2014–2023. Employment status was categorised as: “employed”, “unemployed”, “retired”, “not working due to sickness or disability”, and “other including student, carer or missing”. Housing status was defined as “homeowner”, “renting (private)”, “renting (social housing)”, “rent-free, sheltered accommodation or retirement home”, “unstable (temporary accommodation or homeless)”, or “other/missing”. Financial hardship was assessed by asking “Do you have enough money to meet your basic needs (food, rent etc.)”, with responses: “Yes, always”, “Most of the time”, “Some of the time” and “No”. In our analysis, we defined multimorbidity as reporting ever being diagnosed with two or more of the following conditions in addition to HIV: diabetes, cardiovascular disease (which included heart attack, angina, coronary heart disease, stroke and transient ischaemic attack or ‘mini-stroke’), kidney or renal disease, liver disease, cancer, osteopenia/osteoporosis, arthritis and chronic obstructive pulmonary disease (COPD) (e.g., emphysema or chronic bronchitis). BMI (based on self-reported weight and height) was grouped into four categories: underweight(<18.5 kg/m^2^), healthy weight(18.5–24.9), overweight(25–29.9) and obese(≥30). Smoking status was classed as never, previous or current smokers. Symptoms of depression and anxiety were assessed using PHQ-9 and GAD-7 total scores respectively, using a standard cut-off of ≥10. We used the AUDIT-C screening test score to categorise alcohol consumption as follows: 0 (non-drinkers), 1–2, 3–4, 5–7, 8–10 and 11–12 (possible alcohol dependence).

### Satisfaction with services during COVID

Participants provided their level satisfaction with remote healthcare appointments during the COVID-19 pandemic, including telephone, text messaging and/or via video. Participants were able to respond with “very dissatisfied”, “dissatisfied”, “neutral”, “satisfied”, “very satisfied”, or “not had a remote consultation with this provider” separately for their experiences with their GP, HIV clinics and other health services.

### Statistical analyses

Participants were included in the analysis if they had completed at least one of eight questions in the section on healthcare use in the survey. Among this subgroup, missing data for specific service use was taken to indicate absence of the use of that service. The proportion of people using each healthcare service in the previous year was assessed and variation according to sociodemographic and lifestyle factors were analysed using chi-squared tests for binary and nominal categorical variables and tests for trend for ordered categorical variables. Amongst people who had used each service, we also analysed the distribution of the number of contacts made.

In subsequent analyses, we focused on five key health services of interest: (i) GP and primary health care (ii) A&E (iii) mental health services (iv) sexual health services and (v) dental services. We used modified Poisson regression models with robust standard errors to calculate unadjusted and adjusted prevalence ratios (aPRs) and 95% confidence intervals (95% CIs) to examine associations of sociodemographic and lifestyle factors with the use of each of these key services. This method estimates readily interpretable prevalence ratios instead of odds ratios and is a statistically accepted alternative to logistic regression in the analysis of binary outcomes [19]. However, it should be noted that where the prevalence of the different service use outcomes differ, it is not appropriate to compare the magnitude of prevalence ratios across these different outcomes. In adjusted analyses, each variable of interest was included in a separate model that also included three “core variables” defined prior to any analyses: age group, combined demographic group and year of HIV diagnosis group. All statistical analyses were performed in Stata version 18.0.

## Results

Of the 4622 participants who completed the PV2022 survey, we excluded 88 who did not complete any questions on healthcare service use and a further 83 who did not provide data on age or year of HIV diagnosis. This resulted in 4451 participants included in the analyses (Table 1). Median (interquartile range, IQR) age was 52 (44–60) years, where 2488 (55.9%) were aged of 50 and over and 564 (12.7%) were aged 65 years and over. There were 3315 (74.5%) men, 1,073 (24.1%) women, 31 (0.7%) people with non-binary or other gender and 32 (0.7%) with undisclosed gender. 2893 (65.0%) participants were white, 943 (21.2%) and 131 (2.9%) were Black African and other Black ethnicity respectively, 182 (4.1%) were Asian and 302 (6.8%) mixed or other ethnicity. 2618 (58.8%) participants identified as GBMSM. 476 (10.7%) of people were diagnosed with HIV in 1995 or earlier. 4403 (98.9%) and 4127 (92.7%) self-reported that they were currently on ART and had undetectable viral load (<50 copies/mL) at their last measurement, respectively. 1904 (42.8%) people were educated to at least degree-level and 2731 (61.4%) people were currently in employment. 1848 (41.5%) participants were homeowners while 1015 (22.8%) and 953 (21.4%) lived in private rented properties and social housing respectively. 2213 (49.7%) participants always had enough money to meet their basic needs, whereas 277 (6.2%) never had enough money. 2117 (47.6%) participants accessed their HIV clinic care in London. In our survey population, 684 (15.4%) had two or more comorbidities, 869/4201 (20.7%) had depressive symptoms (PHQ9 ≥ 10) and 627/4184 (15.0%) had symptoms of anxiety (GAD7 ≥ 10). Overall 900/3750 (24.0%) were classed as obese (BMI ≥ 30 kg/m^2^), 699/4035 (17.3%) were current smokers and 1,304/3995 (32.6%) scored ≥5 on the AUDIT-C indicating that drinking may be causing harm to their health.

**Table 1:** Characteristics of the study population (N = 4451).

		N	%
**Age, years**	18-34	344	7.7
	35-44	864	19.4
	45-54	1,325	29.8
	55-64	1,354	30.4
	65+	564	12.7
**Gender**	Men (including trans men)	3,315	74.5
	Women (including trans women)	1,073	24.1
	Non-binary or other gender	31	0.7
	Undisclosed	32	0.7
**Ethnicity**	White	2,893	65.0
	Black African	943	21.2
	Black other	131	2.9
	Asian	182	4.1
	Mixed	136	3.1
	Other (including undisclosed)	166	3.7
**Combined demographic group**	GBMSM	2,618	58.8
	Black African heterosexual men	273	6.1
	Other heterosexual men	363	8.2
	Black African women	598	13.4
	Other women	475	10.7
	Non-binary or other gender	31	0.7
	Undisclosed gender/sexuality	93	2.1
**Year of HIV diagnosis**	2014-2023	997	22.4
	2008-2013	1152	25.9
	2002-2007	1317	29.6
	1996-2001	509	11.4
	1995 or earlier	476	10.7
**Self-reported viral load**	Undetectable (<50 copies/ml)	4127	92.7
	≥50 copies/ml	152	3.4
	Don’t know/missing	172	3.8
**Highest level of education**	Primary or lower secondary education to age 16	969	21.8
	Upper secondary education to age 18 or post-16 qualification	1,010	22.7
	Undergraduate degree	931	20.9
	Postgraduate degree	973	21.9
	Other/missing	568	12.8
**Employment**	Employed	2,731	61.4
	Unemployed	309	6.9
	Retired	569	12.8
	Not working due to sickness or disability	367	8.2
	Other including student or carer/missing	475	10.7
**Housing**	Homeowner	1,848	41.5
	Renting (private)	1,015	22.8
	Renting (social housing)	953	21.4
	Rent-free, sheltered accommodation, retirement home	161	3.6
	Unstable (temporary accommodation, homeless)	43	1.0
	Other/missing	431	9.7
**Money for basic needs**	Yes, always	2,213	49.7
	Most of the time	1,073	24.1
	Some of the time	540	12.1
	No	277	6.2
	Missing	348	7.8
Region of HIV clinic*	London	2,117	47.6
	Midlands and East of England	744	16.7
	North of England	712	16.0
	South of England	827	18.6
	Scotland/Wales/unknown	51	1.1
Comorbidities**	None	2,691	60.5
	1	1,076	24.2
	2 or more (multimorbidity)	684	15.4
**Symptoms of depression**	No	3332	74.9
	Yes (PHQ-9 ≥ 10)	869	19.5
	Missing	250	5.6
**Symptoms of anxiety**	No	3557	79.9
	Yes (GAD-7 ≥ 10)	627	14.1
	Missing	267	6.0
**BMI, kg/m**^**2**^ (self-reported height and weight)	Underweight (<18.5)	78	1.8
	Healthy weight (18.5–24.9)	1,488	33.4
	Overweight (25–29.9)	1,284	28.9
	Obesity (≥ 30)	900	20.2
	Missing/unknown	701	15.7
**Smoking status**	Never smoked	2,027	45.5
	Former smoker	1,309	29.4
	Current smoker	699	15.7
	Prefer not to say/missing	416	9.3
**Alcohol AUDIT-C score**	0 (non-drinker)	1,014	22.8
	1-2 (lowest risk)	882	19.8
	3-4 (low risk)	795	17.9
	5-7 (increased risk)	852	19.1
	8-10 (higher risk)	361	8.1
	11-12 (possible dependence)	91	2.0
	Missing	456	10.2

Some column percentages do not add up to 100% because of rounding.

GBMSM: gay, bisexual and men-who-have-sex-with-men; PHQ-9: Patient Health Questionnaire-9; GAD-7: General Anxiety Disorder-7; BMI: body mass index

*Region: Midlands and East of England (East Midlands, East of England, West Midlands); North (North East, North West, Yorkshire and Humber); South (South East, South West)

**Comorbidities: ever-diagnosed with one or more of diabetes, cardiovascular disease, kidney disease, liver disease, cancer, osteopenia/osteoporosis, arthritis and COPD

Most participants, 98% (4362/4451) were registered with a GP and of these, 89.0% (3883/4362) had shared their HIV status with their GP. Younger people were less likely to have shared their HIV status with their GP compared to older people; 74.6% for ages 18–34 compared to 85.3%, 91.4%, 92.9% and 96.2% in ages 35–44, 45–54, 55–64 and 65 + years respectively. There was little difference between men (89.8%) and women (91.1%) in terms of sharing their HIV status with their GP. People of Asian ethnicity were less likely (81.6%) to have shared their HIV status with their GP compared to people of White (91.3%) or Black African (89.7%) ethnicity.

### Use of health services

**Fig 1:**
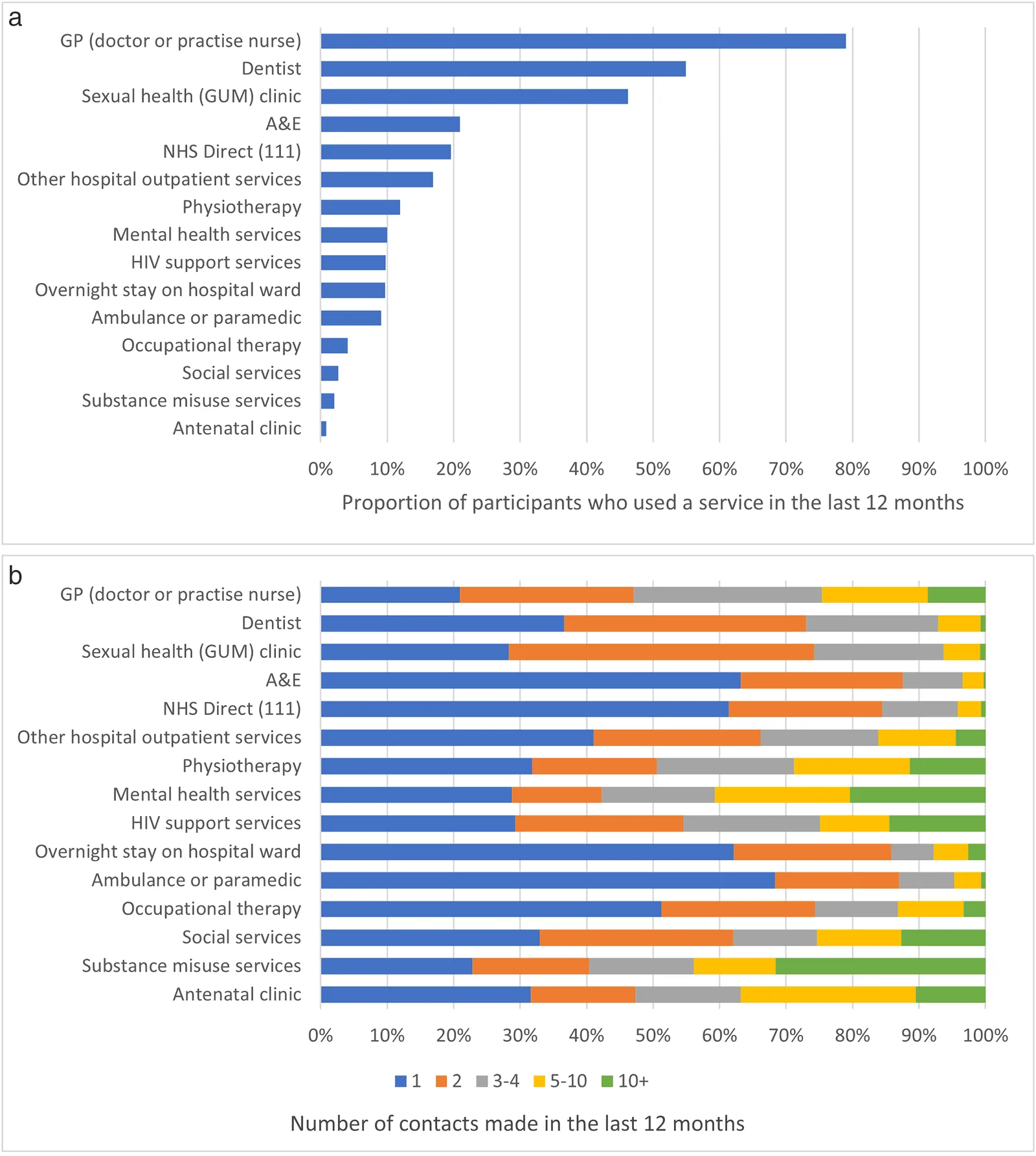
(a) Proportion of people that used NHS health and social care services at least once in the previous year (N = 4451). (b) Frequency of contacts made with each NHS health and social care services in the previous year, as a proportion of the overall number of participants who contacted that service.

The most used health services in the previous year were GP services (79.0% used GPs at least once in the previous year), dentists (54.9%), sexual health clinics (46.2%) and A&E (21.0%) (Fig 1a). 10.0% of people used mental health services in the last 12 months and 9.8% had used HIV support services. For people who used these health services, we also looked at the number of contacts made with each service in the previous year. The median (IQR) number of contacts made with GPs was 3 (2–4) and dentists 2 (1–3). For A&E, mental health and sexual health services, the equivalent figures were 1 (1–2), 3 (1–6.5) and 2 (1–3) respectively. Fig 1b shows the number of contacts with a service as a proportion of the overall use among participants who had used that service. Among users of a specific service in the previous year, frequent use was most common in mental health and substance misuse services. Just over a fifth (20.5%) of mental health services users had used them ten or more times in the previous year. The corresponding percentage for substance misuse service users was 31.6%.

**Fig 2:**
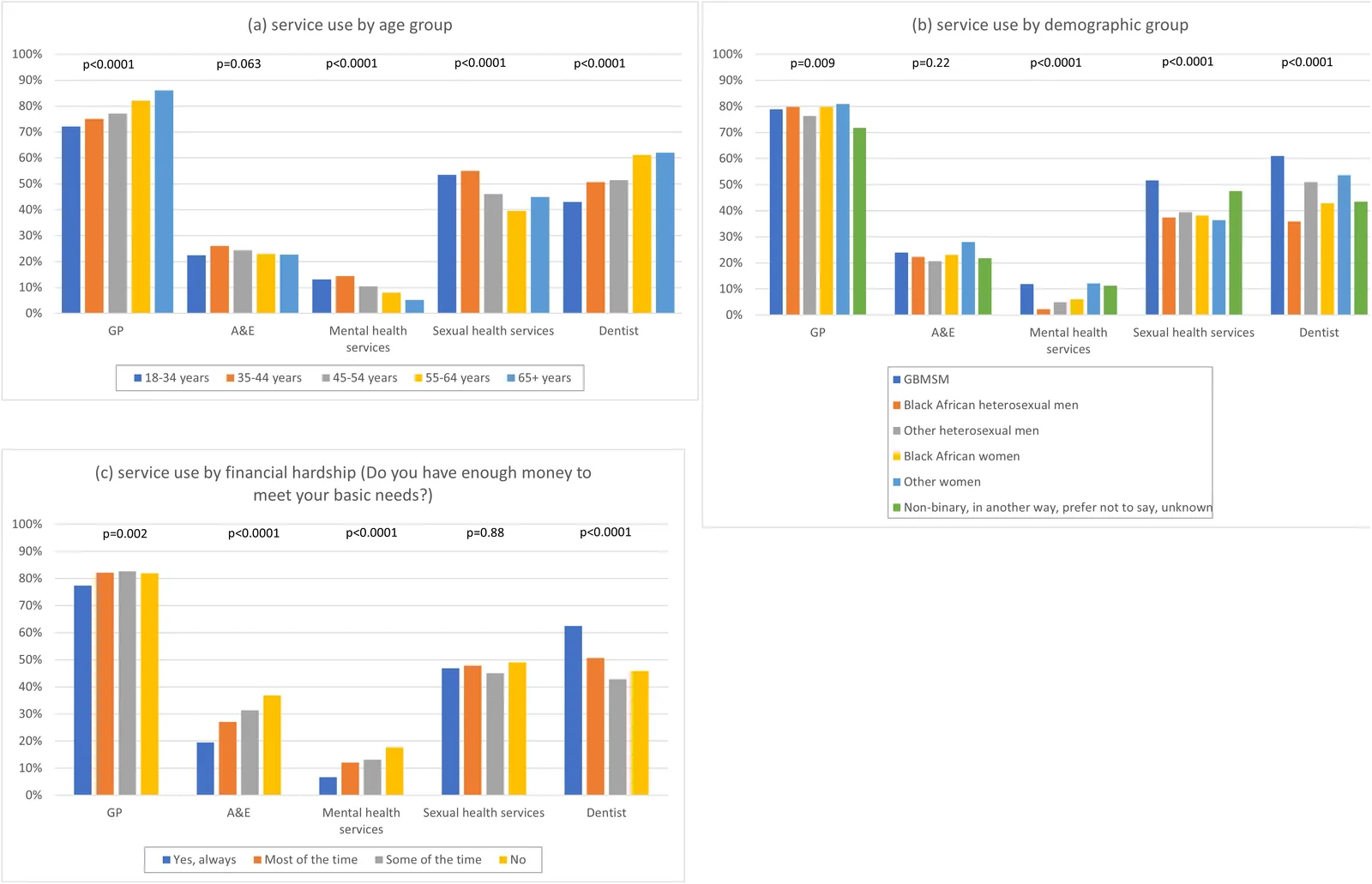
Proportion of participants who reported accessing specific services in the previous year by (a) age group, (b) demographic group and (c) financial hardship (“Do you have enough money to meet your basic needs?”). Footnote: for figs 2a and 2c, p-values are calculated using chi-squared test for trend. For fig 2b, p-value calculated using chi-squared test. For fig 2b, the categories for non-binary or other gender, and undisclosed gender/sexuality were combined due to low numbers.

Fig 2 shows how the proportions of participants using each of the five key services varied by age, demographic group and financial hardship and Table 2 shows the unadjusted and adjusted prevalence ratios.

**Table 2:** Use of healthcare services by sociodemographic factors.

	Unadjusted and adjusted prevalence ratios (95% confidence intervals) and *p-values (calculated using χ*^*2*^ *test)*									
Healthcare service prevalence	GP 79.0%		Accident & emergency 21.0%		Mental health services 10.0%		Sexual health services 46.2%		Dentists 54.9%	
	Unadjusted	Adjusted	Unadjusted	Adjusted	Unadjusted	Adjusted	Unadjusted	Adjusted	Unadjusted	Adjusted
**Combined demographic group**	*P = 0.014*	*P = 0.009*	*P = 0.17*	*P = 0.22*	*P < 0.0001*	*P < 0.0001*	*P < 0.0001*	*P < 0.0001*	*P < 0.0001*	*P < 0.0001*
GBMSM	1	1	1	1	1	1	1	1	1	1
Black African heterosexual men	1.01 (0.95-1.08)	1.02 (0.95-1.08)	0.89 (0.69-1.15)	0.90 (0.70-1.17)	0.18 (0.08-0.41)	0.19 (0.09-0.43)	0.73 (0.62-0.85)	0.73 (0.62-0.85)	0.59 (0.50-0.69)	0.58 (0.49-0.68)
Other heterosexual men	0.97 (0.91-1.03)	0.95 (0.89-1.01)	0.84 (0.66-1.05)	0.86 (0.68-1.08)	0.42 (0.26-0.67)	0.49 (0.31-0.78)	0.76 (0.67-0.87)	0.78 (0.68-0.89)	0.84 (0.75-0.93)	0.81 (0.73-0.90)
Black African women	1.01 (0.97-1.06)	1.03 (0.98-1.07)	0.92 (0.77-1.10)	0.91 (0.76-1.09)	0.51 (0.36-0.71)	0.48 (0.34-0.68)	0.74 (0.66-0.82)	0.72 (0.65-0.81)	0.71 (0.64-0.77)	0.70 (0.64-0.78)
Other women	1.04 (0.99-1.09)	1.04 (1.00-1.09)	1.14 (0.96-1.36)	1.14 (0.95-1.35)	1.02 (0.79-1.33)	1.01 (0.78-1.31)	0.70 (0.62-0.80)	0.70 (0.62-0.79)	0.88 (0.81-0.96)	0.88 (0.81-0.97)
Non-binary or other gender/ undisclosed gender/sexuality	0.91 (0.81-1.02)	0.92 (0.82-1.03)	0.83 (0.56-1.22)	0.83 (0.56-1.21)	0.95 (0.57-1.57)	0.94 (0.57-1.55)	0.92 (0.76-1.12)	0.90 (0.75-1.10)	0.71 (0.58-0.87)	0.74 (0.60-0.90)
**Age (years)**	P < 0.0001*	P < 0.0001*	P = 0.085*	P = 0.063*	P < 0.0001*	P < 0.0001*	P < 0.0001*	P < 0.0001*	P < 0.0001*	P < 0.0001*
18-34	1	1	1	1	1	1	1	1	1	1
35-44	1.05 (0.97-1.12)	1.03 (0.95-1.11)	1.13 (0.89-1.43)	1.15 (0.90-1.48)	1.10 (0.80-1.51)	1.08 (0.77-1.52)	1.03 (0.92-1.15)	1.06 (0.94-1.19)	1.18 (1.03-1.35)	1.14 (0.99-1.31)
45-54	1.07 (0.99-1.15)	1.05 (0.97-1.13)	1.03 (0.82-1.30)	1.04 (0.81-1.33)	0.80 (0.58-1.10)	0.81 (0.57-1.15)	0.86 (0.77-0.97)	0.94 (0.83-1.06)	1.20 (1.05-1.37)	1.18 (1.03-1.35)
55-64	1.14 (1.06-1.22)	1.11 (1.03-1.20)	0.98 (0.78-1.24)	0.97 (0.75-1.24)	0.61 (0.44-0.85)	0.56 (0.39-0.82)	0.74 (0.66-0.83)	0.82 (0.72-0.93)	1.42 (1.25-1.61)	1.36 (1.19-1.55)
65+	1.19 (1.11-1.28)	1.17 (1.08-1.26)	0.91 (0.70-1.20)	0.90 (0.68-1.20)	0.39 (0.25-0.61)	0.35 (0.22-0.57)	0.84 (0.73-0.96)	0.92 (0.80-1.06)	1.44 (1.26-1.66)	1.35 (1.17-1.56)
**Year of HIV diagnosis**	P < 0.0001*	P = 0.017*	P = 0.71*	P = 0.26*	P = 0.51*	P = 0.016*	P < 0.0001*	P < 0.0001*	P < 0.0001*	P = 0.005*
2014-2023	1	1	1	1	1	1	1	1	1	1
2008-2013	1.05 (1.00-1.10)	1.03 (0.98-1.08)	0.91 (0.77-1.08)	0.90 (0.76-1.07)	1.07 (0.84-1.37)	1.16 (0.90-1.51)	0.96 (0.88-1.04)	0.98 (0.90-1.07)	1.16 (1.07-1.26)	1.15 (1.05-1.25)
2002-2007	1.05 (1.01-1.10)	1.02 (0.97-1.07)	0.96 (0.82-1.12)	0.99 (0.83-1.17)	0.82 (0.64-1.06)	1.11 (0.84-1.46)	0.88 (0.81-0.96)	0.97 (0.88-1.06)	1.12 (1.03-1.22)	1.12 (1.03-1.22)
1996-2001	1.10 (1.04-1.16)	1.05 (0.99-1.11)	1.02 (0.84-1.25)	1.09 (0.88-1.34)	0.83 (0.59-1.16)	1.22 (0.85-1.74)	0.83 (0.73-0.93)	0.92 (0.81-1.04)	1.28 (1.17-1.41)	1.19 (1.08-1.32)
1995 or earlier	1.15 (1.09-1.21)	1.09 (1.03-1.15)	1.00 (0.81-1.23)	1.07 (0.85-1.33)	1.09 (0.80-1.48)	1.61 (1.14-2.29)	0.68 (0.60-0.78)	0.74 (0.64-0.85)	1.30 (1.18-1.43)	1.13 (1.02-1.25)
**Employment**	*P < 0.0001*	*P = 0.0001*	*P < 0.0001*	*P < 0.0001*	*P < 0.0001*	*P < 0.0001*	*P = 0.088*	*P = 0.069*	*P < 0.0001*	*P = 0.0043*
Employed	1	1	1	1	1	1	1	1	1	1
Unemployed	1.06 (1.00-1.12)	1.05 (0.99-1.11)	1.64 (1.36-1.97)	1.66 (1.38-2.00)	2.37 (1.81-3.10)	2.63 (2.01-3.45)	0.92 (0.81-1.05)	0.98 (0.86-1.12)	0.84 (0.75-0.95)	0.87 (0.77-0.99)
Retired	1.08 (1.04-1.13)	1.01 (0.95-1.07)	1.01 (0.84-1.22)	1.11 (0.88-1.40)	0.64 (0.44-0.94)	0.98 (0.64-1.50)	0.97 (0.88-1.06)	1.13 (0.99-1.28)	1.19 (1.11-1.27)	1.05 (0.96-1.15)
Not working due to sickness or disability	1.15 (1.11-1.20)	1.13 (1.09-1.18)	1.80 (1.53-2.11)	1.85 (1.56-2.19)	3.49 (2.82-4.32)	3.85 (3.08-4.81)	0.98 (0.87-1.10)	1.09 (0.97-1.22)	1.05 (0.96-1.15)	0.98 (0.89-1.08)
Other including student or carer	0.94 (0.85-1.05)	0.93 (0.84-1.04)	1.22 (0.88-1.68)	1.22 (0.88-1.68)	1.11 (0.62-1.98)	1.22 (0.69-2.19)	0.76 (0.60-0.95)	0.81 (0.64-1.01)	0.68 (0.55-0.85)	0.74 (0.59-0.92)
**Housing**	*P = 0.001*	*p = 0.0001*	*P < 0.0001*	*P < 0.0001*	*P < 0.0001*	*P < 0.0001*	*P = 0.001*	*P = 0.16*	*P < 0.0001*	*P < 0.0001*
Homeowner	1	1	1	1	1	1	1	1	1	1
Renting (private)	1.01 (0.97-1.06)	1.05 (1.01-1.10)	1.34 (1.16-1.55)	1.35 (1.16-1.57)	1.92 (1.50-2.45)	1.79 (1.38-2.31)	1.10 (1.02-1.19)	1.05 (0.97-1.14)	0.70 (0.64-0.75)	0.76 (0.70-0.82)
Renting (social housing)	1.08 (1.04-1.12)	1.08 (1.04-1.12)	1.33 (1.14-1.54)	1.36 (1.16-1.59)	2.28 (1.80-2.90)	2.69 (2.12-3.41)	0.91(0.83-0.99)	1.00 (0.91-1.10)	0.81 (0.75-0.87)	0.87 (0.81-0.93)
Rent-free, sheltered accommodation, retirement home	0.97 (0.89-1.06)	1.00 (0.91-1.10)	1.29 (0.95-1.74)	1.33 (0.98-1.80)	1.63 (0.99-2.67)	1.67 (1.02-2.73)	1.12 (0.96-1.32)	1.10 (0.95-1.28)	0.74 (0.63-0.87)	0.82 (0.70-0.97)
Unstable (temporary accommodation, homeless)	1.04 (0.90-1.21)	1.06 (0.92-1.23)	2.73 (1.98-3.77)	2.99 (2.16-4.14)	4.56 (2.73-7.62)	6.24 (3.76-10.36)	0.85 (0.59-1.24)	0.90 (0.63-1.29)	0.39 (0.24-0.66)	0.48 (0.28-0.81)
**Money for basic needs**	P = 0.001*	P < 0.0001*	P < .0001*	P < 0.0001*	P < 0.0001*	P < 0.0001*	P = 0.88*	P = 0.015*	P < 0.0001*	P < 0.0001*
Yes, always	1	1	1	1	1	1	1	1	1	1
Most of the time	1.06 (1.03-1.10)	1.07 (1.03-1.11)	1.32 (1.15-1.52)	1.37 (1.18-1.58)	1.79 (1.43-2.23)	1.98 (1.58-2.47)	1.03 (0.95-1.10)	1.08 (1.00-1.16)	0.81 (0.76-0.87)	0.85 (0.80-0.91)
Some of the time	1.07 (1.02-1.12)	1.08 (1.03-1.13)	1.56 (1.32-1.83)	1.71 (1.44-2.04)	1.95 (1.50-2.55)	2.61 (1.99-3.42)	0.96 (0.87-1.06)	1.07 (0.96-1.20)	0.68 (0.62-0.76)	0.77 (0.69-0.86)
No	1.06 (1.00-1.12)	1.07 (1.01-1.14)	1.80 (1.48-2.19)	2.00 (1.65-2.45)	2.63(1.95-3.54)	3.41 (2.54-4.59)	1.05 (0.92-1.19)	1.15 (1.01-1.31)	0.73 (0.64-0.84)	0.84 (0.73-0.96)
**Comorbidities**	*P < 0.0001*	*P < 0.0001*	*P < 0.0001*	*P < 0.0001*	*P = 0.033*	*P < 0.0001*	*P = 0.10*	*P = 0.29*	*P < 0.0001*	*P = 0.11*
0	1	1	1	1	1	1	1	1	1	1
1	1.15 (1.11-1.19)	1.13 (1.09-1.17)	1.15 (1.00-1.32)	1.24 (1.08-1.44)	1.28 (1.04-1.57)	1.60 (1.30-1.97)	0.97 (0.90-1.05)	1.06 (0.98-1.15)	1.15 (1.07-1.22)	1.07 (1.00-1.14)
2 or more (multimorbidity)^+^	1.21 (1.17-1.25)	1.18 (1.13-1.23)	1.56 (1.35-1.79)	1.83 (1.56-2.15)	1.25 (0.98-1.59)	1.85 (1.42-2.42)	0.90 (0.82-0.99)	1.06 (0.95-1.18)	1.17 (1.09-1.25)	1.04 (0.97-1.13)
**BMI (kg/m**^**2**^)	P = 0.001*	P = 0.001*	P = 0.082*	P = 0.031*	P = 0.09*	P = 0.096*	P = 0.046*	P = 0.85*	P < 0.0001*	P = 0.10*
Underweight (<18.5)	1.00 (0.89-1.13)	1.00 (0.88-1.12)	0.93 (0.59-1.48)	0.93 (0.58-1.48)	1.88 (1.11-3.17)	1.86 (1.10-3.15)	0.86 (0.67-1.12)	0.89 (0.69-1.14)	1.08 (0.91-1.28)	1.08 (0.91-1.28)
Healthy weight (18.5–24.9)	1	1	1	1	1	1	1	1	1	1
Overweight (25–29.9)	1.03 (0.99-1.07)	1.03 (0.99-1.07)	1.03 (0.89-1.19)	1.06 (0.91-1.22)	1.27 (1.02-1.59)	1.38 (1.10-1.72)	0.92 (0.85-0.99)	0.94 (0.87-1.01)	0.97 (0.91-1.03)	0.98 (0.92-1.04)
Obesity (≥30)	1.08 (1.04-1.13)	1.08 (1.03-1.12)	1.15 (0.98-1.34)	1.20 (1.02-1.41)	1.03 (0.79-1.34)	1.32 (1.00-1.72)	0.91 (0.83-0.99)	1.00 (0.91-1.09)	0.88 (0.81-0.94)	0.95 (0.87-1.02)
**Smoking status**	*P = 0.066*	*P = 0.126*	*P < 0.0001*	*P = 0.0005*	*P < 0.0001*	*P < 0.0001*	*P = 0.32*	*P = 0.89*	*P < 0.0001*	*P = 0.0001*
Never	1	1	1	1	1	1	1	1	1	1
Former smoker	1.04 (1.01-1.08)	1.04 (1.00-1.08)	1.11 (0.97-1.27)	1.12 (0.97-1.29)	1.41 (1.13-1.77)	1.30 (1.03-1.65)	1.04 (0.97-1.12)	1.01 (0.94-1.09)	1.15 (1.09-1.22)	1.04 (0.98-1.10)
Current smoker	1.00 (0.96-1.05)	1.02 (0.97-1.07)	1.40 (1.21-1.63)	1.38 (1.18-1.61)	2.16 (1.72-2.73)	1.79 (1.40-2.30)	1.07 (0.98-1.17)	0.99 (0.90-1.08)	0.93 (0.85-1.01)	0.87 (0.80-0.95)
**Alcohol AUDIT-C score**	*P = 0.02*	*P = 0.029*	*P = 0.095*	*P = 0.052*	*P = 0.02*	*P = 0.005*	*P = 0.005*	*P = 0.40*	*P < 0.0001*	*P = 0.012*
0 (non-drinker)	1.02 (0.97-1.07)	1.01 (0.97-1.07)	1.25 (1.04-1.50)	1.28 (1.06-1.55)	1.50 (1.11-2.03)	1.75 (1.30-2.37)	0.88 (0.80-0.98)	0.95 (0.86-1.05)	0.87 (0.80-0.95)	0.93 (0.85-1.01)
1-2 (lowest risk)	1.07 (1.02-1.12)	1.07 (1.02-1.12)	1.21 (1.00-1.47)	1.22 (1.00-1.48)	1.20 (0.86-1.66)	1.26 (0.91-1.74)	0.95 (0.86-1.05)	0.99 (0.90-1.09)	0.94 (0.86-1.03)	0.95 (0.88-1.04)
3-4 (low risk)	1	1	1	1	1	1	1	1	1	1
5-7 (increased risk)	1.03 (0.98-1.08)	1.04 (0.99-1.10)	1.18 (0.97-1.43)	1.16 (0.95-1.41)	1.51 (1.11-2.07)	1.34 (0.98-1.83)	1.03 (0.94-1.14)	0.99 (0.90-1.09)	1.06 (0.98-1.15)	1.05 (0.97-1.14)
8-10 (higher risk)	1.00 (0.93-1.07)	1.01 (0.95-1.09)	1.09 (0.85-1.41)	1.09 (0.84-1.41)	1.63 (1.12-2.37)	1.52 (1.05-2.21)	1.06 (0.94-1.20)	1.04 (0.92-1.17)	1.07 (0.97-1.18)	1.05 (0.95-1.16)
11-12 (possible dependence)	1.13 (1.04-1.24)	1.14 (1.05-1.24)	1.57 (1.10-2.24)	1.63 (1.14-2.32)	1.96 (1.12-3.43)	2.04 (1.15-3.61)	0.81 (0.62-1.05)	0.80 (0.62-1.04)	0.89 (0.73-1.10)	0.84 (0.69-1.03)

GBMSM: gay, bisexual and men-who-have-sex-with-men; BMI: body mass index; AUDIT-C: Alcohol use disorders identification test consumption

For the combined demographic group variable, we collapsed “non-binary and other gender” and “undisclosed gender/sexuality” into one group due to low numbers

*p-value for test for trend

+ comorbidities included: ever-diagnosed with one or more of diabetes, cardiovascular disease, kidney disease, liver disease, cancer, osteopenia/osteoporosis, arthritis and COPD

(i) **GP service use**

Although GP usage in the past year was high across all groups, there was variation by sociodemographic and lifestyle factors (Fig 2 and Table 2). Patterns of association were similar in unadjusted analyses and after adjusting for the core variables (age, demographic group and year of HIV diagnosis). In terms of demographic factors, GP use tended to be more common among women, and in people aged 55 years or older; adjusted prevalence ratio, aPR:1.11, 95% confidence intervals [1.03–1.20] for 55–64 years and 1.17, 95%CI[1.08–1.26] for 65 years and older compared to 18–34 years respectively. In terms of socioeconomic factors, GP use was more common in people not working due to sickness or disability, people living in rented properties compared to homeowners, and people who did not always have money for basic needs (aPR 1.07, 95% CI[1.01-1.14] for not versus always having money). GP use was also more common in people with comorbidities (aPR 1.18, 95%CI[1.13–1.23] for 2 or more comorbidities versus none), people with obesity, former smokers and people with high levels of alcohol consumption (aPR 1.14, 95%CI[1.05–1.24] for AUDIT-C score 11–12 compared to 3–4).

(ii) **Accident and emergency (A&E) use**

In both unadjusted and adjusted analyses, socioeconomic variables were strongly associated with A&E use in the past year: use was greater in people who were unemployed (aPR 1.66, 95%CI[1.38–2.00]) or not working due to sickness or disability (aPR 1.85, 95%CI[1.56–2.19]) compared to employed people, in people who lived in rented or unstable accommodation compared to homeowners (aPR 1.35, 95%CI[1.16–1.57], aPR 1.36 95%CI[1.16–1.59] and aPR 2.99, 95%CI[2.16–4.14] for private rented, social housing rented and unstable accommodation respectively), and increased in a graded relationship with greater financial hardship (aPR 2.00 95%CI[1.65–2.45]) for people who did not have money to cover basic needs compared to people who always had enough money. Use of A&E was also more likely in people with comorbidity, particularly multimorbidity (aPR [95%CI] 1.83 [1.56–2.15] for 2 or more comorbidities in addition to HIV versus none), in those with obesity, and in current smokers (aPR 1.38 95%CI[1.18–1.61]). In terms of alcohol intake, people who were non-drinkers (scored 0) and those with low (1–2) or very high (11–12) levels of alcohol consumption in AUDIT-C were more likely to use A&E compared to those with intermediate levels (score 3–4). A&E use was not strongly associated with age and was not associated with demographic group or year of HIV diagnosis.

(iii) **Mental health services**

For most variables, patterns of association were similar in unadjusted and adjusted analyses. After adjustment, use of mental health services was considerably lower among Black African men and women as well as other heterosexual men compared to other demographic groups: adjusted PRs for Black African men, other heterosexual men and Black African women compared to GBMSM were 0.19 (95%CI[0.09–0.43]), 0.49 (95%CI[0.31–0.78]), and 0.48 (95%CI[0.34–0.68]) respectively. There was also a strong association with younger age. We found particularly strong associations with socioeconomic factors. Mental health service use was much more likely among people who were unemployed (aPR 2.63, 95%CI[2.01–3.45]) and those not working due to sickness/disability (aPR 3.85, 95%CI[3.08–4.81]) compared to employed, in people who do not own their homes compared to homeowners (aPR 1.79, 95%CI[1.38–2.31], aPR 2.69, 95%CI[2.12–3.41] and aPR 6.24, 95%CI[3.76–10.36] for private rented, social housing rented and unstable accommodation respectively) and increased greatly with greater financial hardship, (aPR 3.41, 95%CI[2.54–4.59] for people who did not have money to cover basic needs compared to people who always had money.

Use of mental health services was also more likely in those outside the healthy BMI range and in previous or current smokers and there was a strong relationship with greater number of comorbidities (aPR 1.85, 95%CI[1.42–2.42])[for 2 or more versus none). Again, there was a U-shaped relationship with alcohol intake, with the highest prevalence of use among people who scored 0 (non-drinker) and 11–12 (very high alcohol consumption) in AUDIT-C and lowest levels in those scoring 3–4. There was an association between earlier year of HIV diagnosis and mental health service use in adjusted analysis only (aPR 1.61 95%CI [1.14–2.29] for diagnosis prior to 1995 versus 2014–2023).

(iv) **Sexual health services**

Use of sexual health services in the past year was greater among GBMSM compared to other demographic groups in both unadjusted and adjusted analyses. Use was also greater among younger people, and lower with earlier calendar period of HIV diagnosis (aPR 0.74, 95%CI[0.64–0.85] for people diagnosed with HIV before 1995 compared to people diagnosed between 2014 and 2023). Socioeconomic factors did not have strong associations with use of sexual health services, though there was a trend of greater use with greater financial hardship. Comorbidity, BMI, smoking and alcohol intake were not associated with use of sexual health services in adjusted analyses.

(v) **Dental services use**

In unadjusted and adjusted analyses, use of dentists in the past year was more likely among GBMSM compared to other demographic groups, with particularly low use among Black African heterosexual men (aPR 0.58, 95%CI[0.49–0.68] vs GBMSM). Use of dental services was more common among with older age, and among people diagnosed with HIV before 2014. The association with socioeconomic factors operated in the opposite direction to that for the other health services: in both unadjusted and adjusted analyses, use of dental services was less likely among unemployed people and those with ‘other’ employment status (student, carer etc), among people living in rented, unstable or rent-free accommodation compared to homeowners and among people who did not always have enough money to cover their basic needs (aPR, 0.84 95%CI[0.73–0.96] for not enough money compared to always enough). Use of dental services was less likely among current compared to never and ex-smokers. Non-drinkers and those with obesity were less likely to use dental services in unadjusted analyses and those with comorbidities were more likely to use dental services; these associations were attenuated in adjusted analysis.

### Remote healthcare consultations

Most people had had a remote consultation with their GP (83.3%, 3241/3893) and HIV clinic (75.9%, 3011/3969) during the period following the COVID-19 pandemic. However, remote consultations were less frequent with other healthcare providers (59.0%, 1837/3114). Satisfaction rates differed greatly between the services, being much higher for HIV clinics compared to other services: 1369/3241 (42.2%), 2200/3011 (73.1%) and 769/1837 (41.9%) of survey participants were either “very satisfied” or “satisfied” with their experience of a remote consultation with their GP, HIV clinician or other healthcare services respectively.

## Discussion

### Summary of findings

Among this national sample of people with HIV, the majority had accessed GP and dental services in the past year, just under half had accessed sexual health services, about one in five had used A&E and one in ten used mental health services, with high frequency of use being particularly common for mental health service users. Use of specific healthcare services outside of HIV care differed by demographic, socioeconomic and lifestyle factors. This may reflect differences in access to services as well as differences in underlying need. Older people were more likely to use GP and dental services, less likely to use mental health and sexual health services, but there was no strong age association with A&E use. Black African men had similar levels of GP and A&E use compared to other demographic groups, but lower use of dental services and much lower use of mental health services. Other heterosexual men and Black African women also had lower use of mental health services. Markers of socioeconomic disadvantage, such as unemployment, rented or unstable accommodation, and not having enough money for basic needs were strongly associated with greater likelihood of use of mental health and A&E services. However, the opposite was true for use of dentists, where people without money to meet basic needs or those living in rented/unstable housing were less likely to access these services. Use of health services also varied according to lifestyle factors. In particular, obesity was associated with use of GP, A&E and mental health services, and current smoking was associated with higher use of A&E and mental health services, lower use of dental services but not associated with GP use. Comorbidity was associated with use of GP and A&E services and particularly strongly with use of mental health services.

### Socioeconomic factors and healthcare service use

It is well established that socioeconomic factors such as education, employment, housing and income are key determinants of health outcomes in the general population [20]. Similar socioeconomic gradients in healthcare use are apparent across many settings with universal access to healthcare [6,7,21], with lower socioeconomic status associated with higher healthcare use. Our findings highlight this socioeconomic association in healthcare service use among people with HIV in the UK, which is especially important given that HIV disproportionately affects socially vulnerable groups [22].

Our analysis showed higher use of A&E and mental health services and to a lesser extent GP use, among those with greater socioeconomic deprivation. Socioeconomic disadvantage is multi-factorial: competing responsibilities, stress, unsettled living circumstances, poor housing and food insecurity may impact on physical and mental health directly or make health a lower priority for the individual, leading to greater levels of ill-health. Further barriers which may inhibit people seeking and attaining healthcare at the earliest opportunity include stigma surrounding poverty, lack of awareness of their need for care and treatment, opportunity costs involved (e.g., time away from zero hours contracts to attend appointments), as well as lack of financial resources or time to attend healthcare services (e.g., transport costs and need for childcare) [23–27].

We found strong socioeconomic gradients for A&E use with increasing use with greater hardship. For example, people who did not always have enough money for basic needs were twice as likely to access A&E compared to people who had enough. This compared to a more moderate gradient for GP use, where the main difference was that those who always had enough money were less likely to have used the GP compared to all other groups. These differing trends may in part reflect delays in seeking timely care for non-HIV related issues for people who have socioeconomic challenges, resulting in greater need to access emergency care. It may also in part reflect disengagement with, or lack of access to, primary care and use of emergency services instead. Research has shown that in the general population, people living in more deprived areas of the UK are more prone to using other NHS services such as 111 and A&E if there are barriers to accessing GP services [28,29]. This effect may be particularly relevant among people with HIV, who routinely access hospital care for their HIV and report higher levels of trust in secondary compared to primary care [30]. Similarly in the current study, people with HIV who were not in employment due to sickness or disability or living in rented or unstable accommodation were much more likely to access A&E compared to those employed or homeowners. We also found that socioeconomic disadvantage was very strongly associated with use of mental health services, with a greater than three-fold increase in prevalence of use across financial hardship groups. This relationship has been described in other studies [31,32] and is related to multiple adverse experiences associated with poverty. Poverty can be both a cause and a consequence of poor mental health. A recent report by the King’s Fund explained that poverty was linked to poorer health outcomes in the UK due to a multitude of complex factors. This included difficulties in accessing healthcare services at an early stage of illness, thereby resulting in later treatment and poorer health outcomes, as well the direct effects on physical or mental health of living in poverty such as those caused by living in unhealthy or overcrowded housing, or inability to afford healthy food or adequate heating [28].

On the other hand, we found significantly lower use of dentists among people experiencing financial hardship as well as those in rented or unstable accommodation and those who were unemployed. An association of socioeconomic deprivation with lower use of dental services is also apparent in the general population [33]. Dental services in the UK are only free of charge to children and the most economically disadvantaged individuals. In addition, there is a significant shortage of NHS dentists in the UK, with many dentists not accepting new NHS patients [34]. Our findings similarly suggest that some people with HIV may be avoiding dental appointments or unable to access dental care due to the costs involved.

### Demographic group and healthcare service use

In our study, we found that Black African heterosexual men were about 80% less likely to use mental health services compared to GBMSM, whereas no statistically significant difference was seen between these two groups accessing GP services and A&E. Other data from PV2022 showed that only 38% of Black African heterosexual men with evidence of a mental health problem were receiving treatment for their condition, compared to 74% of GBMSM [35]. This suggests that lower mental health service use in this group is not solely a feature of lower need, but also reflects disparities in access. Black African men with HIV may be less likely than other groups with HIV to seek mental health services and/or be less likely to be referred to or retained in such services. Lower use of mental health services was also apparent for Black African women and other heterosexual men, who were about half as likely as other women and GBMSM to have used such services. Ethnic inequalities in mental health, and access to mental health services have been recognised as prevalent both among people with HIV and in the general population; people of Black ethnicity are more likely to have undiagnosed and untreated mental illness [36,37] and are less likely to refer themselves or be referred to psychological therapies [38]. A qualitative study from the UK found that ethnic inequalities in access to mental health services may reflect approaches to the assessment and treatment of mental health illnesses which may not fully take into account experiences of racism, migration stress, the role of religion/spirituality and other social circumstances [37]. In addition to these issues, Black African people with HIV may face additional barriers related to stigma that reduce access to and use of mental health services.

### Service use according to lifestyle factors

We also found that smoking and obesity were associated with elevated use of A&E and mental health services. Each of these factors are well known to be preventable causes of increased morbidity and mortality in the general population [39] and may be especially relevant for HIV given the evidence that HIV increases the risk of serious health problems among those who smoke [40,41]. Our results suggest the need to continue to target smoking cessation and to address the high levels of obesity, as smoking and obesity are both estimated to be higher among people with HIV than in the general population, but may also be more prevalent in people with socioeconomic disadvantage [39,41,42].

In our study, alcohol consumption had a complex U-shaped association with some healthcare use outcomes. A&E and mental health service use in particular were highest in the non-drinker or lowest consumption group as well as the very high consumption group as defined by AUDIT-C. Similar U or J-shaped relationships with alcohol use are apparent in the general population for a number of health outcomes [43,44]; higher risk in non or low-drinkers may in part reflect a subset who have stopped drinking due to underlying health/alcohol-related problems.

### Coordination of care and the impact of increased remote consultations

With most non-HIV care managed by services other than HIV clinics, people with HIV have to access and navigate different health and social care services. If services are not well coordinated (“fragmentation of care”) [45] this may add another level of complexity for people with HIV dealing with multiple healthcare services. Recent qualitative work has highlighted the challenges involved for people with HIV. This includes navigating medical appointments in sometimes fragmented health services where structural inequalities may operate, and having the confidence to self-advocate for their needs to be met [46]. Remote consultations were increasingly used during the COVID-19 pandemic [47] and people now have greater choice between remote and face-to-face consultations. However, given the recency of these changes, there is limited data on the impact that remote consultations may have on healthcare use and health outcomes. Other studies have found that since the COVID-19 pandemic, disparities in access to healthcare and health outcomes have further worsened, particularly among people with economic difficulties and for ethnic minorities [48,49].

### Study limitations

PV2022 was a cross-sectional survey in which the use of healthcare services was self-reported so may be subject to responder and recall error or bias. We were unable to differentiate between different types of contact with a healthcare service as a measure of utilisation; an increasing number of consultations and appointments now occur over the phone or online [50] and combining all these contacts together may not be an ideal measure of the quantity of healthcare received. Participants may have had different interpretations of what a contact was: some may have included administrative issues (repeat prescriptions or a need to obtain a referral or letter) and others may have only included consultations. Trends in healthcare use by sociodemographic and lifestyle factors will reflect both variation in underlying need and variation in access to or take-up of services; we were not able to analyse service use controlling for current health need. There are many confounders or mediators such as symptoms of depression and anxiety which we did not fully explore as it was beyond the scope of this analysis. Finally in recruiting for PV2022, although the sample size was large and consisted of nearly 5% of people with HIV in the UK, the study will underrepresent people not accessing regular HIV care. It may also under-represent the most vulnerable groups who were not able to participate or fill out the questionnaire due to illiteracy, language barrier, or lack of time due to other responsibilities. Patterns of non-HIV health service usage may differ in these groups.

## Conclusions

In summary, our findings show that healthcare service use among people with HIV varies considerably across groups defined by demographic, socioeconomic and lifestyle factors, and many of the patterns of use reflect those seen in the general population. In particular, socioeconomic disadvantage was particularly strongly associated with use of A&E and mental health services and was associated with lack of use of dental services.

Development of comorbidities associated with an ageing cohort of people with HIV is likely to require increased use of non-HIV-related healthcare services. This suggests the importance of a multidisciplinary and person-centred approach to ensure timely healthcare service use to support wellbeing and health for each individual. Such an approach would be particularly vital for those with socioeconomic disadvantage. There is also a need for improving provision of support to increase self-management of long-term conditions. In recent years, there has been more research into how to deliver better care for people with HIV through improved integration of care across primary and specialist services [51,52].

In line with findings in the general population, our results also suggest the need to better understand the reasons for marked ethnic disparities in mental health service use among people with HIV in the UK, and in particular to increase access among Black African people. Our findings also emphasise the need to address the underlying causes of disparities in healthcare service use and health outcomes that occur largely due to underlying socioeconomic inequalities; an ongoing and widely acknowledged issue requiring wider societal solutions.

## Supporting information

S1 File PV2022 clinic sites. (DOCX)
