# Supplementary material for: Sociodemographic variations in healthcare service use among people with HIV in the UK
Source: PLoS One. 2026 Sep 15;21(9):e0354425. doi: 10.1371/journal.pone.0354425 (PMC13577419; doi:10.1371/journal.pone.0354425)
Supplement: Supplementary file 1 [file pone.0354425.s001.docx]

| **Hospital** | **PV Site name** |
| --- | --- |
| MORTIMER MARKET CENTRE | Mortimer Market Clinic, London |
| CHELSEA & WESTMINSTER HOSPITAL | Kobler Clinic, Chelsea and Westminster Hospital, London |
| DEAN STREET (GUM) | 56 Dean Street, London |
| ROYAL FREE HOSPITAL | Ian Charleson Day Centre, Royal Free Hospital, London |
| ST MARY'S HOSPITAL (HQ) | Wharfside Clinic, St Mary's Hospital, London |
| THE ROYAL LONDON HOSPITAL | Grahame Hayton Unit, Royal London Hospital, London |
| MANCHESTER ROYAL INFIRMARY | Hathersage Centre, Manchester Centre For Sexual Health |
| ROYAL SUSSEX COUNTY HOSPITAL | Royal Sussex County Hospital, Lawson Unit HIV Clinic, Brighton |
| NORTH MANCHESTER GENERAL HOSPITAL | North Manchester General Hospital, Manchester |
| KING'S COLLEGE HOSPITAL (DENMARK HILL) | Caldecot Centre, Kings College Hospital, London |
| ST GEORGE'S HOSPITAL (TOOTING) | The Courtyard Clinic St George's Hospital, London |
| INFECTIOUS DISEASES SJUH | Brotherton Wing Clinic, Leeds General Infirmary |
| QUEEN ELIZABETH HOSPITAL BIRMINGHAM | Queen Elizabeth Hospital, Birmingham |
| HEARTLANDS HOSPITAL | Birmingham Heartlands HIV Service |
| SOUTHMEAD HOSPITAL | Southmead Hospital, Bristol HIV Service |
| ROYAL LIVERPOOL UNIVERSITY HOSPITAL | Royal Liverpool Hospital, Axess Clinic, Liverpool |
| ALEXIS CLINIC | University Hospital Lewisham, Alexis Unit, Lewisham, London |
| NOTTINGHAM UNIVERSITY HOSPITALS NHS TRUST - CITY CAMPUS | Nottingham Sexual Health Service, Nottingham City Hospital |
| HOMERTON UNIVERSITY HOSPITAL | The Jonathan Mann Clinic, Homerton Hospital, London |
| BARKING HOSPITAL | Barking Community Hospital Sexual Health Clinic, London |
| ROYAL HALLAMSHIRE HOSPITAL | Sexual Health Sheffield, Royal Hallamshire Hospital, Sheffield |
| CROYDON UNIVERSITY HOSPITAL | Croydon University Hospital, Croydon Sexual Health Centre, London |
| 10 HAMMERSMITH BROADWAY CLINIC | 10 Hammersmith Broadway, London |
| QUEEN ELIZABETH HOSPITAL | The Trafalgar Clinic, Queen Elizabeth Hospital, Greenwich, London |
| SEXUAL HEALTH AT BOURNEMOUTH | Sexual Health Dorset, Bournemouth |
| NORTHAMPTON GENERAL HOSPITAL | Northampton General Hospital, Northamptonshire ISHS (HIV) |
| NEWHAM GENERAL HOSPITAL | Newham Hospital, Greenway Centre, London |
| PINDERFIELDS GENERAL HOSPITAL | Wakefield, Spectrum Community Health CIC |
| THE ROYAL SOUTH HANTS HOSPITAL | Royal South Hants, Southampton |
| ROYAL BERKSHIRE HOSPITAL | The Florey Sexual Health Services, Royal Berkshire Hospital, Reading |
| LUTON & DUNSTABLE HOSPITAL | Luton Sexual Health |
| CARDIFF ROYAL INFIRMARY | Cardiff Royal Infirmary |
| SOUTHEND HOSPITAL | Southend Hospital |
| MILTON KEYNES HOSPITAL | Milton Keynes University Hospital, BBV Clinic |
| THE GARDEN CLINIC - P8A | Garden Clinic, Upton Hospital, Slough |
| WITHINGTON CLINIC | Withington Community Hospital, Manchester |
| SEXUAL HEALTH - COBRIDGE | Cobridge Community Health Centre, Stoke-on-Trent |
| C&SH PORTSMOUTH | Portsmouth Sexual Health Service |
| NORFOLK ICASH - OAK STREET | Oak Street Clinic, iCaSH Norfolk, Norwich |
| SIR LUDWIG GUTTMAN CENTRE | Sir Ludwig Guttmann Centre, Stratford, London |
| LONDON ROAD COMMUNITY HOSPITAL | Florence Nightingale Community Hospital, Derby |
| GUM (CHURCHILL) | Churchill Hospital, Oxfordshire Sexual Health Service |
| ADDENBROOKE'S HOSPITAL | Clinic 1a, Addenbrookes Hospital, Cambridge |
| MANOR HOSPITAL | Manor Hospital, Walsall Centre for Sexual Health |
| WATFORD CLINIC | Watford Clinic |
| THE JAMES COOK UNIVERSITY HOSPITAL | The James Cook University Hospital, Middlesborough |
| KINGSTON HOSPITAL | The Wolverton Centre for Sexual Health, Kingston Hospital, London |
| TRINITY ROAD CENTRE | St Lukes Hospital, Bradford |
| WEST MIDDLESEX UNIVERSITY HOSPITAL | Twickenham House, West Middlesex Hospital, London |
| STEVENAGE CLINIC | Stevenage Clinic |
| SURREY SEXUAL HEALTH SERVICE-GUILDFORD | Buryfields Sexual Health Clinic, Surrey Sexual Health Service, Guildford |
| DERRIFORD HOSPITAL | Derriford Hospital, SHiP, Plymouth |
| CAMBRIDGESHIRE COMMUNITY SERVICES - PETERBOROUGH ICASH | iCaSH Peterborough, Peterborough |
| THE GREAT WESTERN HOSPITAL | Swindon Sexual Health Department, The Great Western Hospital |
| ST HELIER HOSPITAL | Rosehill Clinic, St Helier Hospital, London |
| HUDDERSFIELD ROYAL INFIRMARY | The Portland Clinic, Huddersfield Royal Infirmary |
| EXETER (SIDWELL ST) NHS WALK IN CENTRE | Sidwell Street, The Centre, Exeter |
| DEWSBURY & DISTRICT HOSPITAL | Dewsbury Health Centre |
| CRAWLEY HOSPITAL | Crawley Sexual Health, Crawley Hospital |
| EDINBURGH HEALTH BOARD | Chalmers Sexual Health Centre, Edinburgh |
| CHELMSFORD & ESSEX CENTRE | Coelho Clinic, Chelmsford |
| AVENUE HOUSE | East Sussex Sexual Health, Eastbourne Clinic |
| ORWELL CLINIC - CASH | The Orwell Clinic, iCaSH Suffolk, Ipswich |
| MUSGROVE PARK HOSPITAL | Musgrove Park Hospital, The Starling Clinic, Taunton |
| ST MARY'S HOSPITAL [KETTERING] | Summers Unit, Kettering Hospital |
| SEXUAL HEALTH SERVICE [KENT] | Worthing Sexual Health |
| CHESTERFIELD ROYAL HOSPITAL | Chesterfield ISHS |
| ROYAL UNITED HOSPITAL | The Riverside Clinic, Riverside Health Centre, Bath, |
| SEXUAL HEALTH HUB | Clover Street Clinic, Chatham |
| TORBAY HOSPITAL | Devon Sexual Health - Torbay |
| KENT & CANTERBURY HOSPITAL | The Gate Clinic, Canterbury |
| MONKGATE HEALTH CENTRE | Monkgate Health Centre, Sexual Health Clinic York, York |
| KINGS @BECKENHAM BEACON | Beckenham Beacon Sexual Health, Kings College Hospital, London |
| ROTHERHAM DISTRICT GENERAL HOSPITAL | Rotherham General Hospital |
| ST RICHARD'S HOSPITAL | Chichester Sexual Health, St Richard's Hospital |
| WYCOMBE HOSPITAL | Sexual Health at Wycombe, Wycombe |
| SUFFOLK ICASH - CHURCHYARD | Abbey View Clinic, iCaSH Suffolk, Bury St Edmunds |
| BROAD STREET PLAZA | Sexual Health Calderdale, Broad Street Plaza, Halifax |
| SEXUAL HEALTH (STATIONPLAZA) | Hastings Clinic, Station Plaza, East Sussex Sexual Health |
| ST HELENS HOSPITAL | St Helens Hospital Sexual Health (GUM) Service, Wirral |
| OAK TREE CENTRE | Clinic 6, The Oaktree Centre, Huntingdon, iCaSH Huntingdton |
| ICASH - GREAT YARMOUTH | Breydon Clinic, iCaSH Norfolk, Great Yarmouth |
| NORFOLK ICASH - VANCOUVER HOUSE | Vancouver House, iCaSH Norfolk, King's Lynn |
| BROOKSIDE CLINIC | Brookside Clinic, Aylesbury |
| HIV CHESTER UNIT | Fountains Sexual Health Clinic, Chester, |
| ROYAL SHREWSBURY HOSPITAL | Sexual Health Services - Shropshire, Open Clinic, Shrewsbury |
| BISHTON COURT | Bishton Court, Telford |
| WILLIAM HARVEY HOSPITAL | Vicarage Lane Clinic, Ashford |
| QUEEN ELIZABETH THE QUEEN MOTHER HOSPITAL | Newington Road Clinic, Ramsgate |
| SALISBURY DISTRICT HOSPITAL | Salisbury District Hospital |
| BRANSTON HEALTH CENTRE | Branston Clinic, Burton-on-Trent |
| HEATHERDENE | Harrogate Sexual Health Centre |
| SEXUAL HEALTH AT WEYMOUTH | Weymouth Community Hospital, Sexual Health Dorset, Weymouth |
| EAST CHESHIRE SEXUAL HEALTH SERVICE | Macclesfield Hospital, Axess Clinic |
| SEXUAL HEALTH SERVICE [STAFFORD] | Open Clinic, Stafford Clinic |
| BATH STREET HEALTH & WELLBEING CENTRE | Axcess Clinic, Bath Street Health and Wellbeing Centre, Warrington, |
| EAGLE BRIDGE HEALTH AND WELL BEING CENTRE | Axess Clinic, Eagle Bridge Health And Well Being Centre, Crewe |
| NORTHWAY CLINIC | Scarborough Sexual Health Centre, The Mulberry Unit |
| HALTON GENERAL HOSPITAL | Axess Clinic, Halton General Hospital |
| NORTH DEVON DISTRICT HOSPITAL | Devon Sexual Health, Barnstaple |
| ROYAL VICTORIA HOSPITAL FOLKESTONE | Folkestone Health Centre, East Kent HIV Service |
| ST MARY'S HOSPITAL (SEXUAL HEALTH SERVICES) | Sexual Health Service, Isle of Wight |
